# Neurosurgical Intervention in the Treatment of Gradenigo Syndrome: A Case Report

**DOI:** 10.7759/cureus.47573

**Published:** 2023-10-24

**Authors:** Yasmine Rifai, Nicholas Cassimatis, Isaac Soliman MD

**Affiliations:** 1 General Surgery, Hackensack University Medical Center, Hackensack, USA; 2 Neurological Surgery, Hackensack University Medical Center, Hackensack, USA; 3 Internal Medicine, Hackensack Meridian Health Mountainside Medical Center, Montclair, USA

**Keywords:** otitis media, mastoiditis, petrosectomy, abducens nerve palsy, petrous apicitis, kawase approach, gradenigo's syndrome, gradenigo, kawase, neurosurgery

## Abstract

Gradenigo syndrome comprises a clinical triad: retro-orbital pain, sixth cranial nerve palsy, and purulent otorrhea. This clinical syndrome often arises secondary to petrous apicitis, which is an infection of the petrous apex that may result from the contiguous spread of infection from the ear or mastoid. This syndrome is very rare, and based on the existing literature, the initial approach for treatment involves long-term administration of IV antibiotics, which may resolve the underlying infection related to petrous apicitis, mastoiditis, and/or otitis media.

In this case, the patient, a 69-year-old male, had a progression of several symptoms, including recurrent headaches, diplopia, hearing loss, and dysphagia, despite long-term antibiotic therapy and a prior mastoidectomy. Thus, the neurosurgical team decided to intervene via anterior petrous bone resection via the Kawase approach, which unfortunately did not result in the resolution of the patient’s symptoms. The patient continued to have symptoms of Gradenigo syndrome, including sixth cranial nerve palsy and was subsequently referred to outpatient follow-up for further management. In this report, we present the patient’s case and a brief review of the literature concerning various treatment modalities for Gradenigo syndrome.

## Introduction

Gradenigo syndrome is classically defined as a clinical triad consisting of retro-orbital pain, ipsilateral abducens nerve palsy, and purulent otorrhea [1]. However, not all components of this clinical triad are frequently present. Additional presentations include otalgia, fever, coma, and palsy of other cranial nerves, including V, VII, VIII, and X [1]. Symptoms arise due to irritation of the trigeminal ganglion in Meckel’s cave, irritation of the facial, cochlear, or vestibular nerves as they travel through the temporal bone, or paralysis of the abducens nerve as it travels through Dorrell’s canal, which courses through the petrous apex [2]. Gradenigo is a rare syndrome with few reports in the literature and an estimated prevalence of two in 100,000 people.

Gradenigo’s syndrome often arises secondary to petrous apicitis, which is the infection of the petrous apex due to the contiguous spread of infection from mastoid air cell tracts into a pneumatized petrous apex [2,3]. Petrous apicitis may arise as a complication of acute otitis media or mastoiditis and is thus most frequently associated with bacterial etiologies [2]. Other causes of Gradenigo’s syndrome include neoplastic lesions, aspergillus, or nontuberculous mycobacterial infections, though these are far less common [3].

Treatment guidelines for this syndrome recommend the use of long-term IV antibiotics, which is preferred over surgical interventions such as mastoidectomy and labyrinthectomy [3]. This is likely related to the complex anatomy of the petrous apex air cells, which would involve working around the surrounding labyrinth and carotid artery [2]. The following case details an attempt to use petrosectomy in a patient with Gradenigo syndrome to relieve the sixth cranial nerve palsy, which did not seem to relieve his symptoms.

## Case presentation

The patient was a 69-year-old male with a past medical history of poorly controlled diabetes mellitus, petrous apicitis, and septic venous thrombosis with residual sixth and seventh cranial nerve palsies. In June 2022, the patient initiated treatment for a diagnosed malignant otitis externa. However, his condition continued to progress, with symptoms including right hemicranial headache, and he was subsequently found to have right skull base osteomyelitis. In December 2022, he underwent mastoidectomy to treat the osteomyelitis and received ceftazidime via a peripherally inserted central catheter (PICC) line until February. In March 2023, his symptoms returned, and in April 2023, he was readmitted for worsening diplopia, right sixth and seventh nerve palsies, and multiple subclinical seizures arising from the right frontotemporal region. He was found to have meningeal enhancement of the right middle cranial fossa, concerning for basal meningitis. However, a lumbar puncture and blood cultures were unremarkable. An ear swab revealed coagulase-negative Streptococci, and the patient was started on vancomycin, ceftriaxone, and flagyl. During this same admission, he underwent a Kawase approach for anterior petrous bone resection, aiming to resect the petrous apex to improve his sixth nerve palsy.

The decision to approach the patient's syndrome surgically was multifaceted. As mentioned previously, the patient had undergone mastoidectomy five to six months prior to this encounter for skull base osteomyelitis. He had also undergone a prolonged course of antibiotics. His symptoms, including the headache and visual issues, were persistent and progressive in nature despite these treatments. During this admission, ophthalmology was consulted. A lumbar puncture was recommended to evaluate the patient; there was no growth of pathogens, and no abnormalities were revealed. Next, the otolaryngology (ENT) team recommended obtaining imaging and consulting infectious disease and neurology for the patient's abducens palsy. The infectious disease (ID) team was concerned about recurrent right malignant otitis externa with possible osteomyelitis. Due to the patient's intensive antibiotic treatment earlier that year, ID was less concerned about bacterial infection and more suspicious of a fungal or underlying malignancy, given his underlying immunocompromised state from poorly controlled diabetes. Neurology was concerned about a multifactorial, possibly metabolic encephalopathy due to the patient's infection, but also agreed with ID's recommendations.

When the neurosurgical team evaluated the patient, they noted that the patient's MRI revealed inflammation of the petrous apex (unfortunately, these images are unavailable in the medical record). The neurosurgical team had previously published a paper [4] on the utility of the anterior approach to petrous apex resection to improve sixth nerve palsy, and in the context of the patient's symptoms, which have been refractory to antibiotics and previous surgical intervention, they decided to proceed with surgery.

During his operation, the patient underwent a preauricular infratemporal fossa approach to access the petrous apex. Stereotactic image guidance was utilized to register the head in a three-dimensional view, allowing for image-guided resection of the petrous temporal bone. A preauricular curvilinear skin incision was made. A pedicled pericranial innominate fascial flap was harvested and dissected off of the temporalis fascia. Muscle leaflets were reflected to expose the temporal bone. The temporal bone flap was elevated with a match-head bur and drilled down to the temporal squama until the middle fossa was visible. An intraoperative microscope was then utilized to peel the dura propria off the middle fossa from posterior to anterior, and the middle meningeal artery was ligated at the foramen spinosum. The extradural petrous apex lesion was resected by drilling off the anterior petrous bone with a high-speed diamond drill. The horizontal carotid canal was identified and skeletonized to safely drill the rest of the anterior petrous apex. The retrogasserian petrous bone was drilled to decompress the petrous apex, and the central clivus was drilled beyond the inferior petrosal sinus. At this point, the petrosectomy was complete. The free pericranial graft was placed into the petrous apex defect. A cranioplasty of greater than 5 cm was done using a titanium gap plate to reconstruct the temporal squamosal skull base defect. Postoperatively, he was started on broad-spectrum antibiotics, including IV ceftazidime-avibactam, vancomycin, PO fluconazole, and metronidazole. He was then discharged to subacute rehabilitation.

**Figure 1:**
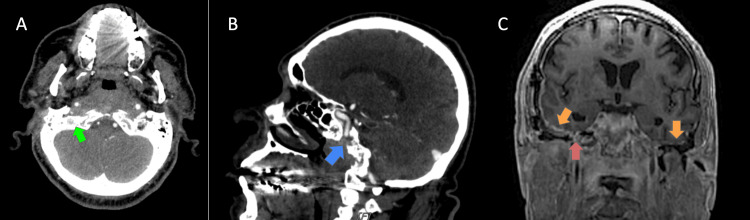
CT head of the patient and MRI of the IACs A: Sclerosis of skull base consistent with sequelae of osteomyelitis (green arrow) B: Right temporal postsurgical changes (blue arrow) with no acute findings C: MRI of the IAC reveals persistent skull base osteomyelitis (red arrow) extending from the right to the left temporal bone, dural thickening and enhancement (orange arrow) along both petrous pyramids and walls of both IACs IAC: Internal auditory canal

In June 2023, the patient returned to the ED with a recurrence of his previous symptoms, including continuous headaches, diplopia, right lateral rectus palsy, slowed heel shin on the left, and progressive hearing loss. He was also experiencing new-onset dysphagia of solid foods. The patient described headaches as bilateral, with shooting pains from the vertex and down both sides of the head and neck. He underwent a CT head, which revealed right temporal postsurgical changes with no acute findings, as seen in Figures 1A-1B. The MRI of the internal auditory canal (IAC), shown in Figure 1C, revealed persistent skull base osteomyelitis extending from the right to the left temporal bone.

The patient was admitted for antibiotic and fungal antimicrobial treatment of his ongoing infection and symptomatic management with ibuprofen and ketorolac. The patient was further evaluated by neurosurgery, ENT, and ophthalmology, and no acute surgical interventions were recommended. Furthermore, the ENT performed cerumen disimpaction, but this did not change the patient's symptoms. The patient was set for an outpatient follow-up with ENT for a medial rectus sheath block and then discharged home.

## Discussion

Gradenigo's syndrome, historically known as Gradenigo-Lannois syndrome, is a rare but clinically significant condition that is characterized by, but not limited to, the classic triad of symptoms: retro-orbital pain, abducens nerve palsy, and purulent otorrhea [1]. Arising chiefly due to infection of the petrous apex, commonly secondary to complications from acute otitis media or mastoiditis, this syndrome is illustrative of how middle ear infections can have extensive neurologic implications. In the modern era of antibiotics, the incidence of intracranial complications secondary to ear infections has substantially declined, with a rate of 2.3% to 6.4% pre-antibiotics and 0.04% to 0.15% post-antibiotics [2]. However, the condition still poses a diagnostic challenge, especially since its presentation can mimic other pathologies such as tumors, vascular issues, or other infections [2].

Early detection and prompt treatment usually lead to a favorable outcome [5]. However, delays in diagnosis or treatment can lead to complications such as meningitis, abscess formation, or further cranial nerve involvement [1]. Regular follow-ups with otolaryngology, neurology, and ophthalmology ensure comprehensive care.

Diagnostic modalities

A clinical examination, such as a thorough otoscopic examination, may reveal signs of otitis media or mastoiditis. Radiographic imaging, such as contrast-enhanced CT or MRI scans, can pinpoint inflammation or infection at the petrous apex [5]. These imaging modalities can also rule out other differential diagnoses. In addition, elevated white cell counts and inflammatory markers might indicate an ongoing infectious process.

Treatment 

Broad-spectrum intravenous antibiotics form the cornerstone of the initial treatment approach, targeting common pathogens responsible for middle ear infections [5,6]. Based on culture results, antibiotics can be de-escalated or tailored as needed. Myringotomy and tympanostomy tubes may be appropriate. In cases where there is significant middle ear effusion, these procedures can facilitate drainage and aid in delivering topical antibiotics [7]. 

Simple mastoidectomy involves the removal of infected air cells in the mastoid bone, providing adequate drainage and access for direct inspection of the middle ear [1]. Radical mastoidectomy, a more extensive procedure, involves the removal of most of the mastoid air cells, tympanic membrane, and middle ear structures, creating an open cavity that connects the external and middle ear [1].

Petrous apicectomy involves the surgical removal of the infected apex of the petrous part of the temporal bone [4]. Given the close proximity of important vascular and neural structures, this is a technically demanding procedure. It allows for the direct drainage of pus and the removal of diseased bone. It is considered when the disease has localized to the petrous apex and does not respond to antibiotics. The Kawase approach is a specific anterior transpetrosal approach, first described by Kawase et al. in 1991 [8]. This approach is primarily used to access lesions in the clivus, petroclival junction, upper clival area, and ventrolateral brainstem. It involves drilling the petrous bone anterior to the IAC and superior to the cochlea, preserving these structures [8]. 

Endoscopic techniques can be employed for better visualization and management, especially for accessing deep-seated regions [4]. The approach can be either transnasal or transmastoid. Rarely, if there is associated hydrocephalus or increased intracranial pressure due to obstruction of cerebrospinal fluid pathways by the infection, ventriculoperitoneal or ventriculoatrial shunting might be considered. Furthermore, while rare for Gradenigo's syndrome, a craniotomy might be necessary for cases that have extensive intracranial complications such as abscesses or significant dural involvement [4].

## Conclusions

In this case, the patient's symptoms due to Gradenigo syndrome and ongoing osteomyelitis were refractory to antibiotic treatment, antifungals, and neurosurgical resection of the impacted portion of his skull. Surgical intervention is typically considered when medical treatment fails or when there are clear surgical indications in Gradenigo syndrome. Furthermore, the choice of a specific surgical approach depends on the extent and location of the disease, the patient's overall health status, and the surgeon's expertise. Additionally, postoperative care, including continued antibiotic therapy and frequent clinical evaluations, is crucial to monitor for resolution or recurrence of the disease. This case is particularly important because it demonstrates the lack of resolution of the patient's symptoms despite exhausting existing and known treatment modalities for Gradenigo syndrome. This further supports the notion that more extensive research and case reports need to be published to explore other treatment options for patients suffering from this syndrome.
